# Relationship between body composition, insulin resistance, and hormonal profiles in women with polycystic ovary syndrome

**DOI:** 10.3389/fendo.2022.1085656

**Published:** 2023-01-09

**Authors:** Haolin Zhang, Wei Wang, Jiaming Zhao, Peijie Jiao, Lin Zeng, Hua Zhang, Yue Zhao, Li Shi, Hangqi Hu, Liyan Luo, Ii Fukuzawa, Dong Li, Rong Li, Jie Qiao

**Affiliations:** ^1^ Department of Traditional Chinese Medicine (TCM), Peking University Third Hospital, Beijing, China; ^2^ Department of Obstetrics and Gynaecology, Peking University Third Hospital, Beijing, China; ^3^ School of Basic Medical Sciences, Peking University, Beijing, China; ^4^ Research Centre of Clinical Epidemiology, Peking University Third Hospital, Beijing, China; ^5^ Center for Reproductive Medicine, Department of Obstetrics and Gynaecology, Peking University Third Hospital, Beijing, China; ^6^ Research Units of Comprehensive Diagnosis and Treatment of Oocyte Maturation Arrest, Chinese Academy of Medical Sciences, Beijing, China

**Keywords:** polycystic ovary syndrome, body fat distribution, waist-to-hip ratio, body fat percentage, insulin resistance, hyperandrogenemia

## Abstract

**Objective:**

To investigate how body fat influences glucose metabolism and hormone profiles in women with polycystic ovary syndrome (PCOS), compared to women without PCOS.

**Methods:**

We conducted a cross-sectional study of 166 women with PCOS and 139 age-matched control women at Peking University Third Hospital (Beijing, China) from March 2016 to December 2021. All participants underwent bioimpedance rate assessment of clinical, anthropometric, hormonal, and metabolic features. In particular, body composition parameters were assessed, based on the methods used in a previous study. Homeostasis model assessment-insulin resistance (HOMA-IR) and other indices calculated from fasting glucose and insulin were used to measure insulin resistance. The hormonal profiles [follicle-stimulating hormone (FSH), luteinizing hormone (LH), estrogen (E2), prolactin (PRL), total testosterone (T), and androstenedione (A2)] were assessed by using biochemical methods. Two subgroup analyses were conducted according to waist-to-hip ratio (WHR; < 0.85, non-central obesity and ≥ 0.85, central obesity) and body fat percentage (BFP; < 35% for lean and ≥35% for obesity). The indices above were analyzed using a two-sided t-test or Wilcoxon rank sum test. Linear regression was used to investigate the effects of body composition on metabolism and sex hormones in the PCOS and control groups.

**Results:**

Compared to women without PCOS, women with PCOS and central obesity (*P*=0.021), PCOS and noncentral obesity (*P*<0.001), PCOS and high BFP (*P*<0.001), and PCOS and low BFP (*P*<0.001) had more severe glucose metabolism evaluated with HOMA-IR. Women with PCOS experienced greater insulin sensitivity impairment than did the normal population for every equal increase in BFP. LH, LH/FSH, total testosterone, and androstenedione were significantly higher in patients with PCOS than in healthy controls, regardless of WHR and BFP stratification. However, negative correlations existed between body fat indices (i.e., BFP and body mass index) and hormone indices (i.e., LH and androstenedione) in the PCOS group, but were absent in the control group.

**Conclusions:**

Obese and non-obese women with PCOS have more severe insulin resistance and sex-hormone disorders than women without PCOS. The effect of body fat on sex-hormone disorders is only exist in women with PCOS. These findings suggested that PCOS clinical guidelines should be more specific to body fat.

**Clinical trial registration:**

https://clinicaltrials.gov/, Registration No. NCT04264832.

## 1 Introduction

Polycystic ovary syndrome (PCOS) is a complex, heterogeneous endocrinopathy in women of reproductive age, with a prevalence of 5%–20% (1) based on the diagnostic criteria (2). The syndrome, in addition to gynecological and hyperandrogenic features, is associated with metabolic abnormalities, which include obesity, dyslipidemia, hyperinsulinemia, and insulin resistance (IR); however, the relationship among these variables remains controversial, as is the link between metabolic abnormalities and hyperandrogenic state (3).

Overweight and obesity are diagnosed in approximately one-half of patients with PCOS and they play a critical role in the development of IR and possibly androgen hypersecretion in these women (4). Obesity, particularly abdominal obesity, is a clinical predictor of high insulin levels, more severe lipid alterations, and increased production of inflammatory substances (5). However, differences also exist in the relationship between IR and hyperinsulinemia in non-obese patients with PCOS. Approximately 40%–50% of patients with PCOS have a body mass index (BMI) in the normal range, and these lean patients with PCOS have an increased risk of metabolic dysfunctions, such as reduced insulin sensitivity with subsequent hyperinsulinemia (6). Individuals with a normal BMI but low body fat percentage (BFP) are at a reduced risk of metabolic anomalies, and the risks are nearly three-fold lower in those with low BFP than those with a high BFP (7). Women with PCOS, especially non-obese women, appear to accumulate more of trunk, body, android, abdominal subcutaneous, and visceral fat compared to BMI-matched controls (8). Therefore, body composition appears to be an important factor in the pathogenesis of PCOS. Obesity intensifies metabolic and possibly hormonal disorders in patients with PCOS, however, whether and to what extent body fat and its distribution changes exist in women with or without PCOS and their correlations with metabolic and reproductive disorders are unclear.

Moreover, BMI has been widely used to define obesity; however, it cannot accurately predict body adiposity (9). Waist circumference (WC) and the waist-to-hip ratio (WHR), which reflect an increase in visceral fat, have been widely used to estimate abdominal obesity in patients with PCOS (10). Anthropometric properties and body composition analyzers are increasingly used to assess obesity and adiposity in clinical and population research.

According to the World Health Organization (WHO) (11), body fat distribution, especially visceral fat and abdominal subcutaneous fat, is a better indicator of metabolic changes than generalized obesity itself, which has been linked to a higher risk of metabolic abnormalities. But some previous investigations defining the parameters of body composition have produced controversial results (12), and the findings inconsistent on whether body fat and its distribution changes exist in healthy women and women with PCOS and their correlations with hyperandrogenism and/or hyperinsulinemia. Compared with controls, more abdominal fat is found in PCOS patients, which may be the cause or early consequence of IR but not hyperandrogenism (6). Other studies have indicated that hyperandrogenism is accompanied by increased intra-abdominal fat storage in PCOS (13). In addition, central fat is strongly associated with a high prevalence of metabolic syndrome, especially cardiometabolic abnormalities. To date, only a few studies have focused on the differential impact of body adiposity levels on the association of metabolic parameters and hormone profiles with fat distribution in patients with PCOS, especially in Asian countries.

Therefore, quantitatively studying body fat deposition in patients with PCOS is essential to obtain in-depth knowledge of their fat distribution. Thus, this study expands on the cross-sectional studies (14, 15) of body composition between PCOS and age-matched normal women to investigate body fat distribution in Chinese women with PCOS and the association of this distribution with metabolic parameters and hormone profiles. Our findings suggest the need to develop clinical guidelines that are specific to body fat, which has an important role in IR. Reducing fat accumulation will therefore aid in the treatment of PCOS.

## 2 Materials and methods

### 2.1 Participants

One hundred and sixty-six women with PCOS and one 139 age-matched healthy control women, aged 18–45 years were included at the clinical department and health center of Peking University Third Hospital (Beijing, China) from March 2016 to December 2021.

#### 2.1.1 Enrollment criteria

##### 2.1.1.1 Inclusion criteria – women with PCOS

PCOS was diagnosed according to the Rotterdam criteria 2003 with at least two of the following symptoms (1): infrequent ovulation or anovulation (2); hyperandrogenism or clinical manifestations of high blood androgen (3); ultrasound findings of polycystic ovaries in one or two ovaries, or ≥ 12 follicles measuring 2–9 mm in diameter, and/or ovarian volume ≥ 10 mL.

##### 2.1.1.2 Inclusion criteria – controls

The control women were healthy, without a history of endocrine disorders, lacked clinical and biochemical evidence of hyperandrogenism (total testosterone < 60 ng/ml, free testosterone < 2 ng/ml, dehydroepiandrosterone sulfate < 271 μg/dl), had regular menstrual cycles occurring every 21–35 days, and had normal ovarian morphology on ultrasonography. They are excluded if they have menstrual irregularities, signs of hyperandrogenism (Ferriman-Gallwey score >4), evidence of PCO morphology on ultrasound.

#### 2.1.2 Exclusion criteria for all women

Individuals were excluded from the study if they had other endocrine disorders, such as androgen-secreting tumors, suspected Cushing’s syndrome, non-classic congenital adrenal hyperplasia (17-hydroxyprogesterone < 3 nmol/L), thyroid dysfunction (TSH<0.55 or >4.78 μIU/ml), hyperprolactinemia (fasting prolactin < 26 ng/ml), type I diabetes or poorly controlled type II diabetes, stage 2 hypertension (resting blood pressure ≥ 160/100 mmHg), psychiatric diagnoses, or use of psychiatric medications, including antidepressants. No women had undergone within 12 weeks any pharmacological treatment (cortisone, antidepressants, antidiabetic treatment such as insulin and acarbose, hormonal contraceptives, hormonal ovulation induction, or other drugs judged at the discretion of the investigator).

### 2.2 Anthropometry measurements

All participants underwent a complete medical examination, including measurements of body weight, height, waist and hip circumference, and blood pressure. Body weight and height were measured at the nearest 0.1 kg and 1 cm, respectively, with an ultrasonic scale (SY-300). BMI was calculated as weight in kilograms divided by the square of height in meters (kg/m^2^). Waist circumference (WC) was measured at the narrowest portion of the torso approximately midway between the lowest rib and the iliac crest, hip circumference (HC) was measured over the widest portion of the gluteal and greater trochanteric region in the standing position, and the WHR was calculated (16).

### 2.3 Body composition assessment

Body composition parameters were assessed based on bioimpedance methods using model MC-180 (Tanita Corporation, model MC-180, Japan (17)), a well-established and validated technique. This measurement was performed during the menstrual period (seven days after expected menses) in the morning. Data obtained included total body fat mass (kg), BFP (%), trunk fat mass (kg), trunk fat percentage (%), and the trunk-to-extremity fat ratio, as previously described.

### 2.4 Biochemical analysis

#### 2.4.1 Insulin resistance

Peripheral blood samples were obtained from all study participants after overnight fasting using a standard venipuncture technique for hormonal and metabolic assessments.

Metabolic profiles assessed were fasting plasma glucose (FPG) and fasting insulin (FINS). Homeostasis model assessment of IR (HOMA-IR) was calculated by using the formula: [FINS (µU/mL) ×FPG (mmol/L)]/22.5; and homeostasis model assessment of β-cell function (HOMA-B) was calculated by using the formula: [FINS (µU/mL) × 20]/[FPG (mmol/L) - 3.5] (18). The Quantitative Insulin Sensitivity Check Index (QUICKI) was calculated by using the following formula: 1/[log FINS (μU/mL) + log FPG (mg/dl)] (19). The fasting glucose-to-insulin ratio (G/I) was calculated by using the formula: FINS (µU/mL)/FPG (mmol/L) (20).

#### 2.4.2 Hormonal profiles

Hormonal profiles including serum follicle-stimulating hormone (FSH, mIU/mL), luteinizing hormone (LH, mIU/mL), estrogen (E2, pmol/L), prolactin (PRL, ng/mL), total testosterone (nmol/L), and androstenedione in nmol/L (A2, nmol/L) were measured by Siemens Immulite 2000 immunoassay system (Siemens Healthcare Diagnostics, Siemens, Germany) (21).

### 2.5 Statistical analyses

The Kolmogorov-Smirnov test and a histogram were used to test whether the continuous variables conformed to a normal distribution. Normally distributed continuous variables were expressed as mean ± standard deviation (SD), and abnormally distributed continuous variables were expressed as medians with interquartile ranges (IQR). The variables were compared between the PCOS and control groups using a two-sided t-test (for normally distributed variables) and the Wilcoxon rank sum test (for abnormally distributed variables). To investigate the role of body composition in PCOS, two subgroup analyses were conducted according to WHR (< 0.85, non-central obesity and ≥0.85, central obesity) (22) and body fat percentage (BFP; < 35% for lean and ≥ 35% for obesity) (23). We compared the differences between the PCOS and WHR/BFP-matched subgroups. Finally, linear regression was used to investigate the varying effects of body composition on metabolism and sex hormone levels in the PCOS and control groups. As an exploratory study, this study did not correct for type I errors. A two-sided P < 0.05 was statistically significant. All statistical analyses were conducted using R software version 4.2.0 (R Foundation, Vienna, Austria. www.r-project.org).

## 3 Results

### 3.1 Participant characteristics

The present study enrolled 166 patients with PCOS and 139 healthy controls for the final analysis. Comparisons of basic demographic, anthropometric, and clinical characteristics between the two groups are shown (**Table 1**). In general, there was no difference in the mean age or height between the PCOS and control groups. Women with PCOS more frequently displayed obesity features, with a larger weight (71.26 ± 14.96 in PCOS, 63.80 ± 12.28 in control, p<0.001), BMI (27.73 ± 5.17 in PCOS, 24.72 ± 4.68 in control, p<0.001), WHR (0.86 ± 0.07 in PCOS, 0.81 ± 0.07 in control, p<0.001), BFP (38.44 ± 7.57 in PCOS, 33.60 ± 7.91 in control, p<0.001), trunk fat percentage (39.32 ± 9.33 in PCOS, 33.39 ± 9.87 in control, p<0.001), trunk-to-extremity fat ratio (1.16 ± 0.16 in PCOS, 1.08 ± 0.21 in control, p<0.001), as well as WC, HC, total body fat mass and trunk fat mass than the control population.

**Table 1:** Participant characteristics^a^.

Characteristic	PCOS	Control	*P* value
	(n=166)	(n=139)	
	(Mean ± SD)	(Mean ± SD)	
Age, y	29.44 ± 5.80	29.45 ± 8.22	0.987
Height, m	1.60 ± 0.06	1.61 ± 0.05	0.336
Weight, kg	71.26 ± 14.96	63.8 ± 12.28	**<0.001**
Body mass index, kg/m^2^	27.73 ± 5.17	24.72 ± 4.68	**<0.001**
Waist circumference, cm	89.13 ± 12.7	80.93 ± 12.2	**<0.001**
Hip circumference, cm	103.22 ± 9.33	98.94 ± 8.61	**<0.001**
Waist-to-hip ratio	0.86 ± 0.07	0.81 ± 0.07	**<0.001**
Total body fat mass, kg	28.44 ± 11.14	22.33 ± 9.23	**<0.001**
Body fat percentage, %	38.44 ± 7.57	33.6 ± 7.91	**<0.001**
Trunk fat mass, kg	15.41 ± 6.49	11.83 ± 5.61	**<0.001**
Trunk fat percentage, %	39.32 ± 9.33	33.39 ± 9.87	**<0.001**
Trunk to extremities fat ratio	1.16 ± 0.16	1.08 ± 0.21	**<0.001**
Fasting plasma glucose, (mmol/L)	5.1 (4.7;5.6)	5.1 (4.8;5.4)	0.522
Fasting insulin, (µU/ml)	14.05 (9.6;21.06)	8.7 (5.2;13.21)	**<0.001**
HOMA-IR	3.07 (2.14;5.07)	2.02 (1.17;3.2)	**<0.001**
HOMA-B	181.19 (119.35;268.92)	109.33 (71.83;157.78)	**<0.001**
QUICKI	0.32 (0.3;0.34)	0.34 (0.32;0.37)	**<0.001**
G/I	0.36 (0.25;0.54)	0.57 (0.41;0.98)	**<0.001**
Prolactin, ng/mL	10.95 (8.54;14.68)	12.7 (9.8;17.55)	0.018
Follicle-stimulating hormone, mIU/mL	5.84 (4.8;7.02)	6.07 (5.12;7.06)	0.299
Luteinizing hormone, mIU/mL	7.03 (3.67;9.52)	4.23 (3.17;5.2)	**<0.001**
LH/FSH	1.12 (0.67;1.75)	0.72 (0.47;0.88)	**<0.001**
Estrogen, pmol/L	180 (137.5;225.75)	169 (143;204)	0.168
Total testosterone^b^, nmol/L	1.02 (0.69;1.49)	0.69 (0.69;0.81)	**<0.001**
Androstenedione, nmol/L	11.55 (7.34;16.17)	7.1 (5.38;9.57)	**<0.001**

^a^ Data conforming to the normal distribution were expressed as mean ± standard deviation, and the bilateral t-test was applied. Data that did not conform to a normal distribution were expressed as median (lower quartile, upper quartile), and the bilateral Wilcoxon rank-sum test was applied. ^b^ The lower detection limit for total testosterone was 0.69. HOMA-IR: homeostasis model assessment of insulin resistance; HOMA-B: homeostasis model assessment of β-cell function; QUICKI: quantitative insulin sensitivity check index; G/I: fasting glucose to insulin ratio. P-values with statistical differences (P < 0.05) have been bolded.

In addition, women with PCOS presented with significantly higher levels of FINS [14.05 (9.60;21.06) in PCOS, 8.70 (5.20;13.21) in control, p<0.001], HOMA-IR [3.07 (2.14;5.07) in PCOS, 2.02 (1.17;3.20) in control, p<0.001], and HOMA-B [181.19 (119.35;268.92) in PCOS, 109.33 (71.83;157.78) in control, p<0.001] and lower levels of QUICKI [0.32 (0.30;0.34) in PCOS, 0.34 (0.32;0.37) in control, p<0.001] and G/I [0.36 (0.25;0.54) in PCOS, 0.57 (0.41;0.98) in control, p<0.001] than control participants.

Additionally, we did not observe any differences in the levels of serum PRL, FSH, or E2. Interestingly, women in the PCOS group had higher serum LH [7.03 (3.67;9.52) in PCOS, 4.23 (3.17;5.20) in control, p<0.001], LH/FSH [1.12 (0.67;1.75) in PCOS, 0.72 (0.47;0.88) in control, p<0.001], total testosterone [1.02 (0.69;1.49) in PCOS, 0.69 (0.69;0.81) in control, p<0.001], and A2 [11.55 (7.34;16.17) in PCOS, 7.1 (5.38;9.57) in control, p<0.001] concentrations.

### 3.2 Subgroup analysis

To characterize relationship between body fat and various body parameters, we performed several subgroup analyses.

#### 3.2.1 Waist-to-hip ratio subgroup analysis

For the WHR subgroup analysis, the PCOS and control groups were stratified using a WHR of 0.85. WHR ≥ 0.85 was defined as central obesity and WHR < 0.85 was defined as non-central obesity (24). The PCOS group had a WHR of 0.86 ± 0.07, which was significantly higher than 0.81 ± 0.07 in the control group (p < 0.001). The rate of central obesity in the PCOS group (53.61%) was significantly higher than that in the control group (31.65%; p < 0.001).

As shown in **Table 2**, the central obese population had no significant differences in body size or composition in the PCOS group, compared to the control group, except for the WHR. However, in the non-central obese population, all listed body composition indicators in the PCOS group were significantly higher than those in the control group. For example, among the non-central obese population, the PCOS group, compared to the control group, had a significantly higher BMI (25.25 ± 4.76 vs. 22.76 ± 3.95; *P*<0.001) and BFP (34.89% ± 7.52% vs. 30.32% ± 7.04%; *P*<0.001).

**Table 2:** Comparison of body composition indices of patients with PCOS and controls after waist-to-hip ratio stratification^a^.

Body composition indicators	Central obesity			Non-central obesity		
	PCOS (n=89)	Control (n=44)	*P* value	PCOS (n=77)	Control (n=95)	*P* value
Weight, kg	77.11 ± 14.17	74.27 ± 8.85	0.159	64.5 ± 12.93	58.95 ± 10.52	**0.003**
Body mass index, kg/m^2^	29.87 ± 4.53	28.96 ± 3.06	0.175	25.25 ± 4.76	22.76 ± 3.95	**<0.001**
Waist circumference, cm	96.55 ± 10.98	93.76 ± 6.6	0.071	80.55 ± 8.45	74.99 ± 9.27	**<0.001**
Hip circumference, cm	105.83 ± 9.06	104.64 ± 6.32	0.379	100.21 ± 8.75	96.29 ± 8.26	**0.003**
Waist-to-hip ratio	0.91 ± 0.05	0.9 ± 0.04	**0.041**	0.8 ± 0.03	0.78 ± 0.04	**<0.001**
Total body fat mass, kg	32.82 ± 10.8	30.52 ± 6.82	0.139	23.38 ± 9.27	18.54 ± 7.61	**<0.001**
Body fat percentage, %	41.52 ± 6.17	40.67 ± 4.23	0.355	34.89 ± 7.52	30.32 ± 7.04	**<0.001**
Trunk fat mass, kg	18.03 ± 6.21	16.85 ± 3.87	0.183	12.38 ± 5.42	9.5 ± 4.7	**<0.001**
Trunk fat percentage, %	43.06 ± 7.6	42.13 ± 5.08	0.405	34.99 ± 9.33	29.34 ± 8.88	**<0.001**
Trunk to extremities fat ratio	1.21 ± 0.12	1.24 ± 0.12	0.251	1.09 ± 0.17	1 ± 0.21	**0.002**

a The distribution was expressed as mean ± standard deviation, and a bilateral t-test was applied. P-values with statistical differences (P < 0.05) have been bolded.

**Figure 1:**
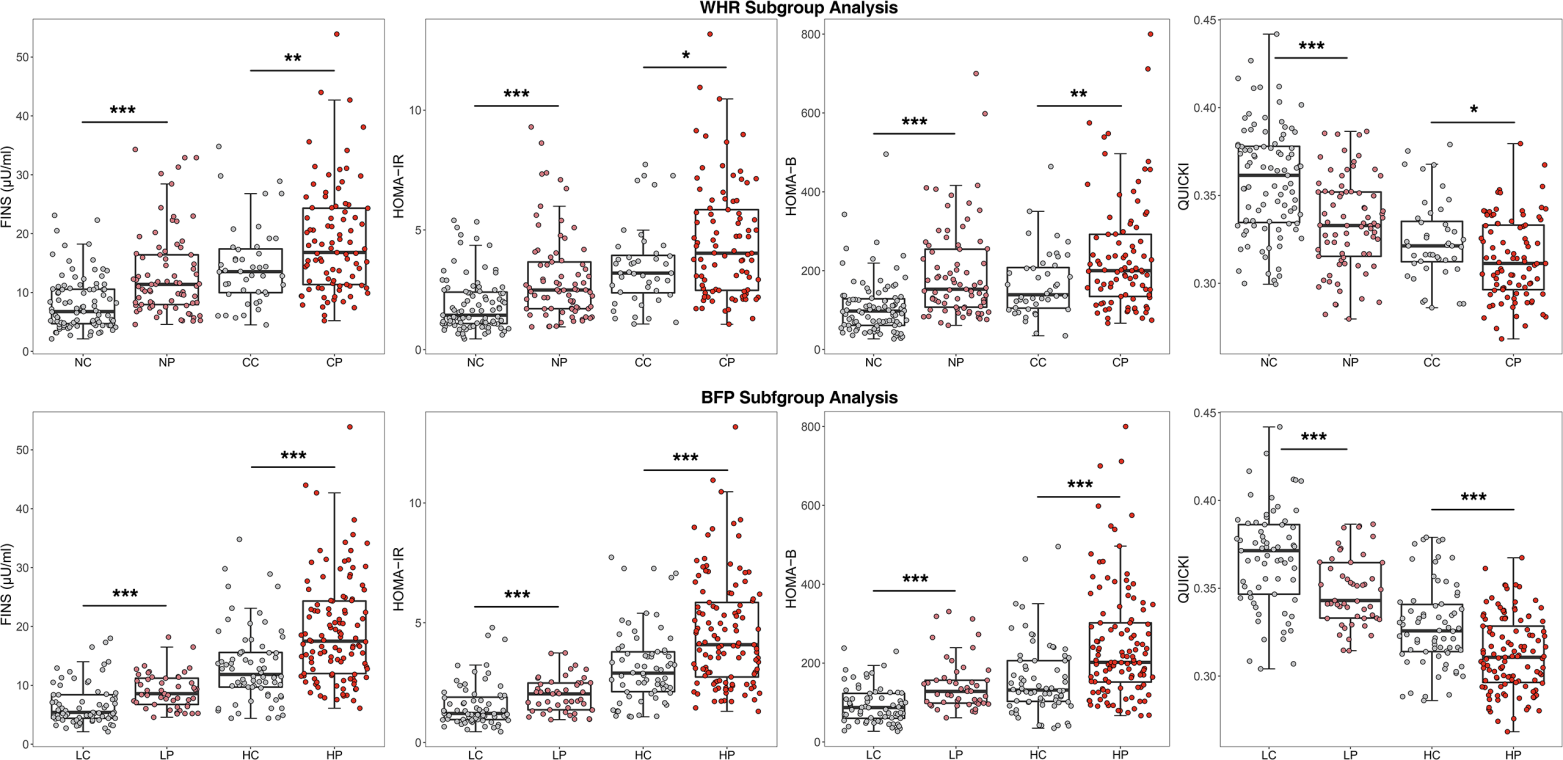
Comparison of glucose metabolism indicators after WHR and BFP stratification^a^.^a^WHR, Waist-to-hip ratio; BFP, Body fat percentage; CP, Central obese PCOS group; CC, Central obese control group; NP, Non-central obese PCOS group; NC, Non-central obese control group; HP, High body fat percentage PCOS group; HC, High body fat percentage control group; LP, Low body fat percentage PCOS group; LC, Low body fat percentage control group; FINS, fasting insulin; HOMA-IR, homeostasis model assessment of insulin resistance; HOMA-B, Homeostasis model assessment of β-cell function; QUICKI, quantitative insulin sensitivity check index. * p < 0.05, ** p < 0.01, *** p < 0.001.

Regarding glucose metabolism. As shown in **Figure 1**, regardless of the obesity status, the PCOS group had more severe glucose metabolism impairment than the control group. For example, among the central obese population, HOMA-IR was 4.03 (2.48;5.85) in the PCOS group and 3.20 (2.38;3.94) in the control group (*P*=0.021), among the non-central obese population, HOMA-IR was 2.49 (1.71;3.66) in the patients with PCOS and 1.44 (1.09;2.41) in the control group (*P <*0.001). Moreover, insulin levels and HOMA-B in the PCOS group were significantly higher than those in the control group, and QUICKI and G/I in the PCOS group were significantly lower than those in the control group. In addition, the difference between the PCOS and control group was more pronounced among the noncentral obese participants than among the central obese participants.

Considering that BMI and BFP were higher in the non-central obese PCOS group than in the control group, we also performed multiple linear regression to adjust BMI and BFP to investigate the differences in glucose metabolism between PCOS and control groups with non-central obesity. As shown in **Table 3**, HOMA-IR, HOMA-B, QUICKI, G/I in the PCOS group were still significantly higher than those in the control group after adjusting for BMI and BFP, consistent with findings without the adjustment.

**Table 3:** Comparison of PCOS and Control glucose metabolism in non-central obesity after adjustment for body mass index and body fat percentage^a^.

Glucose metabolism indicators	Non-central obesity			
	PCOS(n=77)	Control (n=70)	*P* value(Before adjustment)	*P* value(After adjustment)
Fasting plasma glucose, (mmol/L)	4.8 (4.6;5.5)	5.1 (4.8;5.3)	0.156	**0.017**
Fasting insulin, (µU/ml)	11.38 (7.95;16.4)	6.76(4.7;10.56)	**<0.001**	**<0.001**
HOMA-IR	2.49 (1.71;3.66)	1.44(1.09;2.41)	**<0.001**	**0.002**
HOMA-B	152.91 (107.44;254.44)	97.5 (61.25;128.64)	**<0.001**	**<0.001**
QUICKI	0.33 (0.32;0.35)	0.36 (0.33;0.38)	**<0.001**	**<0.001**
G/I	0.47 (0.29;0.6)	0.72 (0.49;1.08)	**<0.001**	**<0.001**

^a^ Variables were expressed as medians with interquartile ranges. Both the interaction term between group and body mass index (BMI) and the interaction term between group and body fat percentage(BFP) were not significant. Only the main effects of group, BMI and BFP were examined, and the p values of all regression equations were less than 0.001. HOMA-IR: homeostasis model assessment of insulin resistance; HOMA-B: homeostasis model assessment of β-cell function; QUICKI: quantitative insulin sensitivity check index; G/I: fasting glucose to insulin ratio. P-values with statistical differences (P < 0.05) have been bolded.

**Figure 2:**
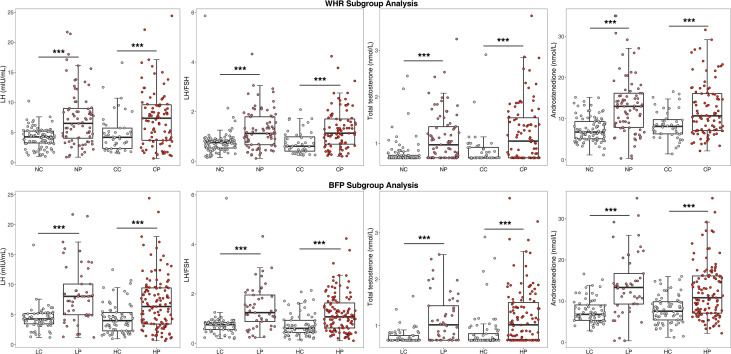
Comparison of endocrine indicators after WHR and BFP stratification^a^.^a^ WHR, Waist-to-hip ratio; BFP, Body fat percentage; CP, Central obese PCOS group; CC, Central obese control group; NP, Non-central obese PCOS group; NC, Non-central obese control group; HP, High body fat percentage PCOS group; HC, High body fat percentage control group; LP, Low body fat percentage PCOS group; LC, Low body fat percentage control group; FSH, follicle stimulating hormone; LH, luteinizing hormone. * p < 0.05, ** p < 0.01, *** p < 0.001.

With regard to endocrine hormones, the level of LH was 7.35 (3.67, 9.59) mIU/mL in the central obese PCOS group, 4.16 (2.30, 5.71) mIU/mL in the central obese control group, 6.49 (4.02, 8.99) mIU/mL in the noncentral obese PCOS group, and 4.24 (3.22, 5.20) mIU/mL in the noncentral obese control group. Regardless of the presence of central or noncentral obesity, the PCOS group, compared to the control group, generally had significantly increased LH, LH/FSH, total testosterone, and androstenedione levels, but had no significant differences in the levels of TSH, PRL, FSH, and E2 (**Figure 2**).

#### 3.2.2 Body fat percentage subgroup analysis

The BFP was 38.44% ± 7.57% in the PCOS group, which was significantly higher than the 33.60 ± 7.91% in the control group (*P*<0.001). Based on the WHO standard, the critical value of BFP for obesity is 35% for adult women. Therefore, the PCOS and control groups were stratified by the BFP of 35%. A BFP ≥35% was defined as a high BFP and a BFP <35% was defined as a low BFP. The proportion of participants with a high BFP in the PCOS group (70.48%) was significantly higher than that in the control group (50.36%, *P*<0.001).

As shown in **Table 4**, in the population with a high BFP, the PCOS group, compared to the control group, had significantly higher values for the following features: WC (94.25 ± 10.99 vs. 91.11 ± 7.02; *P*=0.018), total body fat mass (33.31 ± 9.40 vs. 29.85 ± 6.34; *P*=0.003), BFP (42.19 ± 5.20 vs. 40.29 ± 3.90; *P*=0.005), trunk fat mass (18.30 ± 5.37 vs. 16.45 ± 3.65; *P*=0.006), and trunk fat percentage (43.96 ± 6.35 vs. 41.69 ± 4.69; *P*=0.006). However, no significant differences existed in HC, WHR, or trunk-to-extremity fat ratio. In the population with a low BFP, nearly all the body composition indicators, including BMI, WC, HC, WHR, total body fat mass, BFP, trunk fat mass, trunk fat percentage and trunk-to-extremity fat ratio in the PCOS group, were significantly higher than those in the control group, except for weight. For example, in the low BFP population, the following indicators were significantly higher in the PCOS group than in the control group: BMI (21.98 ± 2.32 vs. 20.73 ± 2.09; *P*=0.003) and WHR (0.81 ± 0.05 vs. 0.76 ± 0.04; *P*<0.001)

**Table 4:** Comparison of body composition indexes of PCOS and control groups after body fat percentage stratification^a^.

Body composition indicators	High body fat percentage			Low body fat percentage		
	PCOS (n=117)	Control (n=70)	*P* value	PCOS (n=49)	Control (n=69)	*P* value
Weight, kg	77.59 ± 12.47	73.41 ± 8.46	**0.007**	56.16 ± 7.95	54.05 ± 6.49	0.131
Body mass index, kg/m^2^	30.14 ± 3.99	28.67 ± 2.8	**0.003**	21.98 ± 2.32	20.73 ± 2.09	**0.003**
Waist circumference, cm	94.25 ± 10.99	91.11 ± 7.02	**0.018**	76.9 ± 6.8	70.61 ± 6.13	**<0.001**
Hip circumference, cm	106.86 ± 8.37	105.34 ± 6.19	0.158	94.54 ± 4.53	92.43 ± 5.14	**0.021**
Waist-to-hip ratio	0.88 ± 0.06	0.87 ± 0.05	0.058	0.81 ± 0.05	0.76 ± 0.04	**<0.001**
Total body fat mass, kg	33.31 ± 9.4	29.85 ± 6.34	**0.003**	16.8 ± 4.08	14.71 ± 3.87	**0.006**
Body fat percentage, %	42.19 ± 5.2	40.29 ± 3.9	**0.005**	29.5 ± 4	26.81 ± 4.34	**0.001**
Trunk fat mass, kg	18.3 ± 5.37	16.45 ± 3.65	**0.006**	8.52 ± 2.49	7.14 ± 2.47	**0.004**
Trunk fat percentage, %	43.96 ± 6.35	41.69 ± 4.69	**0.006**	28.23 ± 4.92	24.97 ± 5.71	**0.001**
Trunk to extremities fat ratio	1.22 ± 0.11	1.23 ± 0.12	0.509	1.01 ± 0.15	0.92 ± 0.18	**0.004**

a The distribution was expressed as the mean ± standard deviation, and a bilateral t-test was applied. P-values with statistical differences (P < 0.05) have been bolded.

With regard to glucose metabolism and endocrine hormones, the results of BFP stratification were generally consistent with those of WHR stratification, as shown in **Figures 1** and **2**. In the high BFP population, HOMA-IR in the PCOS group [4.08 (2.74, 5.85)] was significantly higher than that in the control group [2.90 (2.12, 3.79), *P*<0.001]; among the low BFP population, HOMA-IR in the PCOS group [2.03 (1.37, 2.48)] was significantly higher than that in the control group [1.22 (0.96, 1.89), *P*<0.001]. Consistent with HOMA-IR, whether BFP was high or low, the FINS and HOMA-B values in patients with PCOS were significantly higher than those in the control group (*P*<0.001), and the QUICKI and the G/I ratio were significantly lower than those in the control group (*P*<0.001). The LH level was 6.35 (3.44, 9.45) mIU/mL in the high BFP PCOS group, 4.00 (2.30, 5.34) mIU/mL in the high BFP control group, 8.04 (4.98, 10.10) mIU/mL in the low BFP PCOS group, and 4.28 (3.46, 5.20) mIU/mL in the low BFP control group. Regardless of BFP stratification, the LH level, the LH/FSH, total testosterone, and A2 levels were significantly higher in the PCOS group than in the control group (*P*<0.001), but the TSH, PRL, FSH, and E2 levels were not significantly different.

### 3.3 Regression analysis

**Figure 3:**
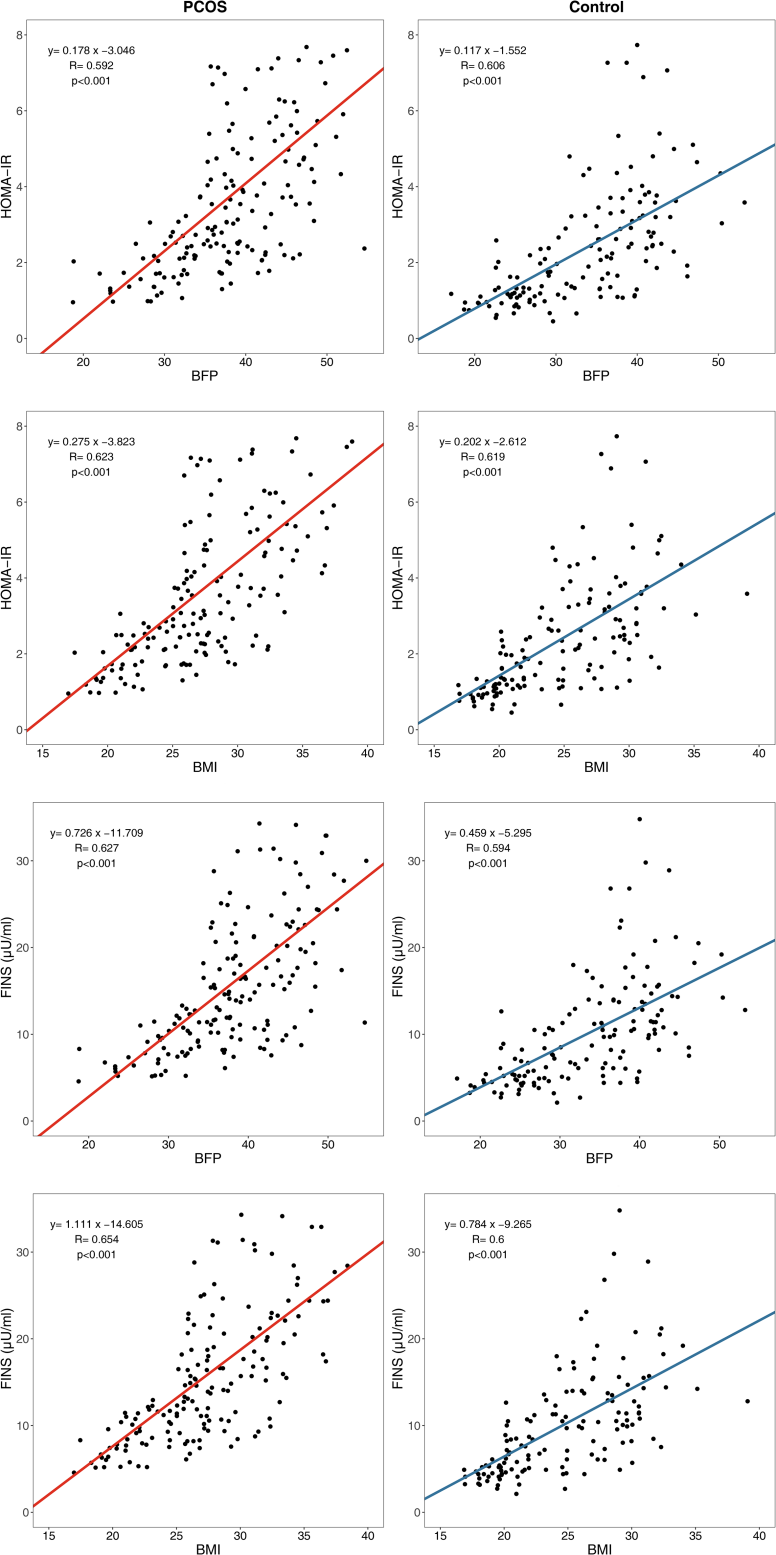
Linear regression between body composition index and glucose metabolism index. HOMA-IR, homeostasis model assessment of insulin resistance; FINS, fasting insulin; BFP, body fat percentage; BMI, body mass index.

An analysis of the linear regression between the body composition index and glucose metabolism index showed that the β regression coefficient between HOMA-IR and BFP was higher in the PCOS group (β= 0.178, *P*<0.001) than in the control group (β= 0.117, *P*<0.001) (**Figure 3**), which was statistically significant (*P*=0.010). In addition, the β regression coefficient between the other glucose metabolism indicators and other body composition indexes such as HOMA-IR and BMI (*P*=0.046), FINS and BFP (*P*=0.003), and FINS and BMI (*P*=0.020) showed the same results.

**Figure 4:**
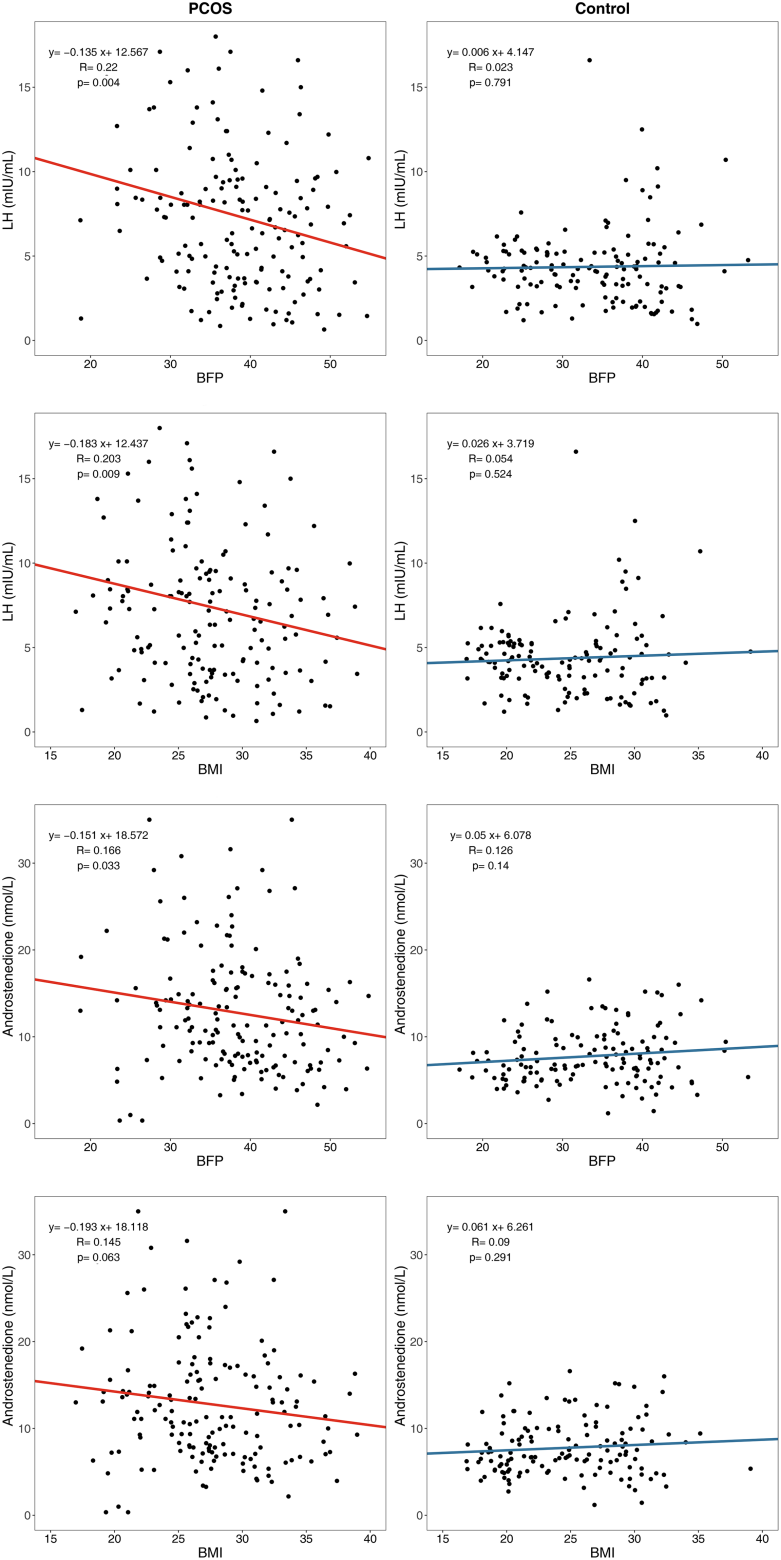
Linear regression between body composition index and endocrine index. LH, Luteinizing hormone; BFP, Body fat percentage; BMI, body mass index.

The body composition and endocrine indices were weakly correlated in patients with PCOS. However, the LH levels (β = –0.135, P=0.004) and androstenedione levels (β = –0.151, P=0.033) decreased slightly with increasing BFP and showed a weak negative correlation in the PCOS group; this correlation did not exist in the control group (**Figure 4**).

## 4 Discussion

The aim of this study is to investigate how body fat influences glucose metabolism and hormone profiles in women with PCOS, compared to women without PCOS. We found that obese and non-obese women with PCOS have more severe insulin resistance and sex-hormone disorders than women without PCOS. Furthermore, we conducted a major comparison between healthy and PCOS women and performed subgroup analysis under different patterns of BMI, WHR and BFP, suggesting that although some PCOS women have normal BMI or WHR, their body fat compositions are still higher than those in healthy women, providing a new understanding of the body fat distribution for PCOS. Additionally, our results manifest that impaired glucose metabolism is present not only in PCOS patients with central obesity and high BFP, but also in those with normal WHR and BFP. It provides evidence for the necessity to investigate the body fat and metabolic disorders in all PCOS population and to assess daily interventions and clinical treatments. Meanwhile, these results show that levels of androstenedione and LH correlated with fat accumulation, which has not been sufficiently examined in previous studies and deserves further investigation.

PCOS is the most common endocrine, metabolic, and psychological disorder in women of reproductive age. Women with PCOS are more likely to be central obese and elevated risk of metabolic disturbance. Some conflicting results may result from the bias from different measurement methods (8). Moreover, the body fat deposition may be completely different in PCOS women with the same BMI (8). Consistent with the results of previous studies (25–27) that report an elevated prevalence of central obesity estimated with the WC in women with PCOS, the present study demonstrated that among Chinese people, the central obesity rate of patients with PCOS (53.61%, estimated by WHR) is higher than the rate in controls (31.65%). Patients with PCOS have a more obese body shape higher accumulation of fat, higher WHR, and higher trunk/periphery fat ratio, representing central obesity. In addition, the BFP cut-offs for obesity have been proposed by the WHO as 35% for women and 25% for men (23). The obesity rate of patients with PCOS (70.48% estimated by BFP) is higher than that of the controls (50.36% estimated by BFP).

A notable finding was that a subgroup analysis, stratified by WHR, showed that non-central obese patients with PCOS exhibited greater total body fat mass, BFP, trunk fat mass, and trunk fat percentage than did the noncentral obese controls. Taking the WHR subgroup analysis as an example, the BFP and trunk fat percentage in the non-central obese patients with PCOS were 34.89% ± 7.52% and 34.99% ± 9.33%, respectively, which were higher than 30.32% ± 7.04% and 29.34% ± 8.80% in the non-central obese controls. This result indicated that, although the BMI and WHR of some patients with PCOS were in the normal range, their body fat compositions were still higher than those of healthy people, which suggested that fat accumulation also exists in patients with non-central obese PCOS.

The fact that obesity has a critical role in the development of IR and adverse metabolic outcomes in patients with PCOS has been widely acknowledged (4, 5). A survey in the United States found that the prevalence of IR in patients with PCOS was 64%-80% (28). In the present study, 58.43% of patients with PCOS and 32.37% of controls had IR. Findings of the FINS, HOMA-IR, QUICKI, G/I, and other IR indicators suggested that glucose metabolism disorders were more severe in PCOS patients, compared to the controls. Furthermore, after conducting subgroup analysis, stratified by WHR and BFP, the FINS and HOMA-IR values were higher in patients with PCOS than in the controls, and the QUICKI and G/I ratio values were lower in patients with PCOS than in the controls. These results were consistent across all subgroups (i.e., the central obesity, noncentral obesity, high BFP, and low BFP subgroups). This finding indicated the possible presence of other pathogenic factors of PCOS, besides obesity, that lead to hyperinsulinemia and IR. In addition, glucose metabolism is impaired not only in PCOS patients with central obesity and high BFP, but also in PCOS patients with a normal WHR and BFP. This finding is consistent with our findings and that of Kirchengast and Huber (29), which revealed significant differences in body composition and fat distribution between lean women with PCOS and lean controls. Excessive accumulation of adipose tissue may reduce insulin sensitivity by secreting adipokines and activating proinflammatory cytokines, thereby resulting in these differences. As a result, a recommendation is that patients with PCOS with noncentral obesity and normal BFP undergo regular examinations of glucose metabolism.

Linear regression analysis showed that the β regression coefficient between HOMA-IR and BFP is higher in the PCOS group than in the control group, which indicated more severely impaired insulin sensitivity in patients with PCOS than in the controls for every equal increase in BFP. This finding suggested that some pathogenic factors of PCOS, acting as modifying factors, may accelerate the pathophysiological pathway of lipid-induced glucose metabolism impairment.

However, the β regression coefficients between HOMA-IR and WHR had no statistical significance when comparing PCOS group and control group (*P* =0.872), so did the β regression coefficients between FINS and WHR (*P* =0.973). Although the results of WHR stratification were generally consistent with those of BFP stratification in subgroup analysis, WHR could not reflect PCOS glucose metabolism impairment compared with normal population in linear regression analysis as BFP did. As a result, BFP may be relatively more sensitive than WHR in predicting abnormal glucose metabolism in PCOS patients.

For endocrine disorders, the Wilcoxon rank-sum test showed similar levels of FSH in the PCOS group and the control group. However, the LH and LH/FSH levels in the PCOS group were significantly higher than those in the control group, suggesting that an increase in the LH level is the main manifestation of gonadotropin metabolism disorder in patients with PCOS. In addition, total testosterone, and A2 levels were significantly higher in patients in PCOS than those of the controls. This finding may be related to the abnormal frequency and amplitude of GnRH release from the hypothalamus in patients with PCOS, which leads to an increase in pituitary LH release and stimulates androgen production. These results are independent of the WHR and BFP subgroup analyses, thereby implying that sex hormone disorders are less affected by obesity.

Although the levels of LH and androstenedione in patients with PCOS were higher than those in the controls, the LH and androstenedione levels decreased slightly with an increase in BFP and BMI in patients with PCOS, showing a weak negative correlation which did not exist in the control group. This result is consistent with that of previous studies showing that LH levels are significantly lower in women with PCOS with a BMI >25 kg/m^2^ (30) and a BMI >24 kg/m^2^ (31) than in women with a normal BMI. These results suggest that obesity may be a modifying factor in sex hormone disorders instead of the cause of sex hormone disorders. This finding implied that patients with PCOS with different BFP and BMI values may have different PCOS pathogeneses. Our previous study (32) explored the influence of fat distribution on PCOS phenotypes in a rat model and indicated that gonadotropic dysfunction and IR may influence PCOS phenotypes through distinct mechanisms; thus, raising the possibility that PCOS could be differentiated into subcategories. For women with PCOS who have normal BFP and BMI, the main cause of PCOS symptoms may be gonadotropic dysfunction, whereas for women with PCOS who have high BFP and BMI, the cause may be IR.

However, the weak negative correlation between LH/androstenedione and BFP/BMI should be further established. In addition, we did not collect data on free testosterone. Due to the limitations of instrumental detection methods, the lower limit of total testosterone detection is 0.69 nmol/L, which is not suitable for linear regression analysis. Then, we applied logistic regression, but failed to examine correlation between total testosterone and BFP in PCOS (*P*=0.582) and control (0.101), and correlation between total testosterone and BMI was not detected in PCOS (*P* =0.490) and control (*P*=0.136) either. In the future, we will continue to collect more androgen indicators and conduct more detailed studies on the relationship between body composition indicators and androgen indicators. Finally, our study’s findings indicated that body fat may have an important role in IR in women with PCOS, which demonstrates the need to develop clinical guidelines for the management of PCOS that takes into consideration patients’ body fat. Nevertheless, we should note the limitations of the study is that it had a cross-sectional design. Thus, only correlational rather than causal conclusions can be drawn. The study did not explore all relevant indicators such as sex hormone-binding globulin and lipid metabolism indices; hence, further study is needed to explore the underlying mechanisms of fat content in PCOS.

## 5 Conclusion

Our findings suggested that body fat plays a major role in determining IR in women with PCOS, who had more insulin sensitivity impairment than did the normal population for every equal increase in BFP, which emphasizes the importance of weight management in patients with PCOS. However, patients with PCOS and noncentral obesity should not be ignored because they should also undergo regular examinations for glucose metabolism. The results of the study will aid clinicians and healthcare professionals in designing more effective treatment plans and clinical guidelines for patients with PCOS.

## Data availability statement

The raw data supporting the conclusions of this article will be made available by the authors, without undue reservation.

## Ethics statement

This study was approved by the Regional Ethical Review Board of the Peking University Third Hospital (PKU3-IRB- 2016-212-02). The patients/participants provided their written informed consent to participate in this study.

## Author contributions

HLZ and WW designed and organized this study, obtained ethics and data protection approval, collected study objects, provided data management and wrote this manuscript. JZ and PJ conducted the statistical analysis, took responsibility for the integrity and accuracy of this analysis, interpreted the data and drafted the manuscript. LZ, HZ participated in study design, methodology and statistical analysis. YZ, LS and LL collected, acquired and coordinated the data. HH, LL and IF drafted the manuscript, interpreted findings and critically revised this manuscript. DL, RL and JQ revised the paper, supervised this project and contributed to the expert review and survey instrument. All authors have read and approved the final manuscript for submission and publication, and agree to be accountable for all aspects of the work. All authors contributed to the article and approved the submitted version.
